# 

**Published:** 1887-07

**Authors:** 


### BOOK NOTICES.

The Esoteric, Vol. i. No. i of this new and progressive monthly is before us, and we heartily welcome it, as the harbinger of "A New Cycle of Progress," after the title of its leading article, which has a strong leaning toward the most ancient of the sciences, astrology, and announces that the earth is just entering upon a state of spiritual growth, paralleled only by a similar state 25,824 years ago, from which it relapsed to its former state of ignorance and indifference of spiritual truth. But at that early period, it is to be presumed that the means of acquiring and retaining knowledge, as compared with the present, were greatly deficient. But with the manifold means of to-day of preserving and handing down to future generations all that has been gained in the whole range of letters, it is scarcely possible for the world to relapse into a darkness corresponding to that which overspread it in the middle ages, and buried, as in a tomb, so many of the ornaments of a past and almost forgotten civilization.

Publications of the class of *The Esoterie* are a gain, in every sense worth considering.

For Boys.—This is the title of a most instructive volume of four hundred pages, containing a wide range of information upon the organic structure of plants and animals, including the laws of generation, preservation and health, adapted to the comprehension and needs of youth. It is indeed a very complete physiology, aptly and amply illustrated, with some chapters upon adult manhood, marriage, and the family relation, which it would benefit cvery young person to read and profit by.

One of the objects of this work is to show that all the processes of nature are interestingly beautiful; that it is only to the coarse and vulgar minded that they are made to appear otherwise. It is indeed the *concealment*--the unwise suppression of some of the essential laws of life—which often times couples their tardy and uninformed recognition with feelings akin to shame, as if it were possible for nature to offend the rules of propriety, in the operation of laws which gave to every one existence, and the capacity of procreation.

This book will do much toward correcting an evil which has its outgrowth in ignorance and misconception, as many other social evils have. It is by no means a too early visitor, and should find an open door to the homes of the youth of all classes.

Published by the Sanitary Publishing Co., Chicago, and sent by mail, postpaid, at $2.00.

The Youth's Companion.—The enterprise of this interesting weekly is unabated. This week's issue contains an original essay by Professor Huxley, F. R. S., entitled "From the Hut to the Pantheon," besides an abundance of other interesting matter, with many apt illustrations. Perry Mason & Co., Publishers, Boston.

Horticultural Art Journal, Part 6. present series of this excellent monthly is on our table. The illustrations are apples, grapes and gooseberries about the size of grapes, colored to life. Published by Stecker Lithographic Co., Rochester, N. Y.

Spelling.—The above is the comprehensive name of a new magazine devoted to the arduous task of simplifying English authography. It is the official organ of the Spelling Reform Associations, among whose list of officers and members may be found the names of many of the foremost scholars of this country and Europe. As a reform

Journal we wish it success in its mission and the support it asks. Price $1 per year, Publishing Library Bureau, 32 Hawley St,, Boston, Mass.

SOCIAL SCIENCE.—A new comer under the above title makes its debut under date of June 24th. Its leading article is a biographical sketch of Albert K. Owen the projector of the Topolobampo co-operative settlement in Sinaloa Mexico, about which, much has been said and written of late pro and con "Free from dogma, independent and radical" are the watchwords of its salutatory: On that line we give it welcome. Published weekly, at 3, East 14th. St. N. Y. City.

